# KRT8 upregulation promotes tumor metastasis and is predictive of a poor prognosis in clear cell renal cell carcinoma

**DOI:** 10.18632/oncotarget.19198

**Published:** 2017-07-12

**Authors:** Hai-Song Tan, Wei-Hua Jiang, Yi He, De-Sheng Wang, Zhen-Jie Wu, Deng-Shuang Wu, Li Gao, Yi Bao, Jia-Zi Shi, Bing Liu, Li-Jun Ma, Lin-Hui Wang

**Affiliations:** ^1^ Department of Urology, Changzheng Hospital, Second Military Medical University, Shanghai 200003, China; ^2^ Department of Oncology, Shanghai Tongren Hospital, Shanghai Jiaotong University, Shanghai 200336, China; ^3^ Department of Urology, Jiaxing First Hospital, Zhejiang 314000, China; ^4^ Department of Urology, Second People's Hospital of Bengbu City, Anhui 233000, China; ^5^ Department of Pathology, Changhai Hospital, Second Military Medical University, Shanghai 200433, China

**Keywords:** clear cell renal cell carcinoma, metastasis, KRT8, IL-11, biomarker

## Abstract

Keratin 8 (KRT8) plays an essential role in the development and metastasis of multiple human cancers. However, its role in clear cell renal cell carcinoma (ccRCC) remains unexplored. Here, we investigated the expression pattern, clinical significance, and function of KRT8 in ccRCC. KRT8 mRNA and protein levels were determined in two large cohorts using quantitative real-time polymerase chain reaction (qRT-PCR) and tissue microarray (TMA) immunohistochemistry (IHC), respectively. We found that KRT8 expression was upregulated in ccRCC and vein tumor thrombi (VTTs). KRT8 overexpression in ccRCC was significantly correlated with aggressive characteristics and was predictive of a poor prognosis in ccRCC patients. Moreover, KRT8 overexpression in renal cancer cell lines promoted cell migration and invasion. In contrast, KRT8 knockdown suppressed ccRCC metastasis both *in vitro* and *in vivo*. In addition, our findings showed that KRT8 promoted ccRCC metastasis by increasing IL-11 expression, causing IL-11 autocrine induction, and triggering STAT3 signaling. Overall, this study established the significance of KRT8-IL-11 axis activation in aggressive ccRCC and defined a novel critical signaling mechanism that drives human ccRCC invasion and metastasis.

## INTRODUCTION

Renal cell carcinoma (RCC) has historically been one of the most common and aggressive urological malignancies [1]. Of all the RCC histological subtypes, clear cell renal cell carcinoma (ccRCC) is the most prevalent and invasive and is associated with high rates of mortality and resistance to chemotherapy and radiotherapy [2, 3]. Although great progress has been made with respect to the diagnosis and treatment of ccRCC, approximately 30% of newly diagnosed patients and 20% to 40% of postoperative patients will still experience metastasis or local recurrence of their disease [4–7]. In the event of tumor metastasis, the five-year survival rate in affected patients is only 0-13% [8]. Unfortunately, compared with other tumors, there are few effective biomarkers for ccRCC [9]. Thus, it is essential for us to elucidate the molecular mechanisms underlying ccRCC progression and metastasis and identify specific biomarkers that can be used for predicting clinical prognosis and developing novel therapeutic approaches for the treatment of patients with ccRCC.

Keratins (KRTs) account for the majority of intermediate filament (IF) proteins and are preferentially expressed in epithelial cells [10, 11]. According to their biochemical characteristics, including their molecular weights and isoelectric points, keratin proteins can be divided into type I acidic and type II basic proteins [12–14]. KRTs are essential components of the cytoskeleton and are involved in various cellular processes, such as differentiation, mitosis, and apoptosis [15]. For example, KRT19 expression has been reported to be associated with poor tumor differentiation and aggressive tumor behavior in hepatocellular carcinoma [16]. Elevating KRT13 expression can directly drive metastasis to bone tissue, brain tissue and soft tissue in prostate cancer [17]. In cervical cancer, high KRT17 expression is correlated with advanced tumor stages and poor patient prognosis [18], and KRT17 loss induces tissue-specific cytokine polarization and effector immune cell recruitment to foster the creation of a protective environment for lesion-prone cervical tissue [19].

KRT8, a type II basic intermediate filament protein, often paired with KRT18, is expressed in many simple epithelial cells, such as hepatocytes, pancreatic acinar and islet cells, and proximal tubular kidney epithelial cells [15]. Obermajer and colleagues [20] found that KRT8 combined with urokinase-type plasminogen activator, plasminogen and fibronectin constituted a signaling platform that can modulate breast tumor cell adhesion and invasiveness. Increased KRT8 expression conferred resistance to cadmium-induced apoptosis and tumor necrosis factor (TNF)-induced apoptosis and thus accounted for cadmium-induced carcinogenesis [21]. KRT8 was positively expressed in head-and-neck squamous cell carcinomas and metastases, but not in hyperplastic leukoplakia, indicating that KRT8 can be used as an attractive marker molecule for differentiating between diagnoses of leukoplakia and head-and-neck carcinomas [22, 23]. Fang et al. [24] reported that high KRT8 mRNA and protein expression was correlated with human gastric cancer progression and metastasis in tumor tissues and that KRT8 expression may be used as a prognostic biomarker for gastric cancer patients. Furthermore, a recent study provided evidence that KRT8 can confer resistance to apoptosis in granulosa cell tumors by impairing FAS expression [25]. However, the role of KRT8 in ccRCC pathogenesis remains unknown. Therefore, in this study, we evaluated the expression pattern, clinical significance and biological function of KRT8 in ccRCC.

## RESULTS

### KRT8 upregulation in primary metastatic ccRCC tissues

**Figure 1:**
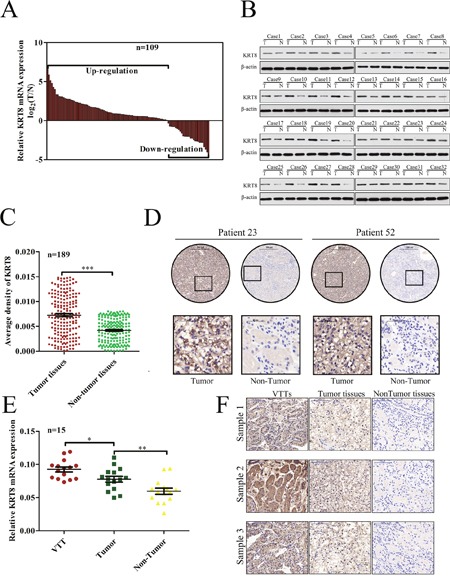
KRT8 upregulation in primary metastatic ccRCC tissues **(A)** KRT8 mRNA expression levels in 109 paired ccRCC and adjacent NT tissues were evaluated by qRT-PCR. **(B)** Western blots showing KRT8 protein expression in tumor tissues (T) and paired adjacent NT tissues (N) from 32 ccRCC patients. **(C)** Relative IHC staining for KRT8 expression in paired ccRCC tissue samples (n=189). KRT8 expression levels were significantly upregulated in tumor tissues compared with corresponding adjacent non-tumor renal tissues. **(D)** IHC characteristics of KRT8 in ccRCC and adjacent NT tissues. Representative staining results for KRT8 are shown. **(E)** Relative KRT8 expression levels in 15 pairs of ccRCC and VTT tissues, as assessed by qRT-PCR. **(F)** Representative IHC images of matched VTT/tumor/peritumor tissues.

To clarify the significance of KRT8 expression in ccRCC, we evaluated KRT8 mRNA and protein levels in fresh frozen ccRCC tissues and adjacent NT tissues. In the qRT-PCR cohort including 109 pairs of ccRCC tissues and adjacent NT tissues, KRT8 expression was markedly upregulated in the ccRCC tissues of most patients (74.31%) (Figure 1A). The Western blot results showed that KRT8 protein levels were also upregulated in ccRCC tissue samples compared with adjacent NT renal tissue samples (Figure 1B). We subsequently performed IHC for KRT8 expression using ccRCC tissue microarrays containing 189 paired ccRCC samples. As shown in Figure 1C, the staining density of the KRT8 protein in the tumor group was stronger than that in the peritumor group (average KRT8 protein density: 0.0072±0.00031 vs 0.0042±0.00017; P<0.0001; paired t test). Representative IHC staining results are shown in Figure 1D. A previous work showed that advanced-stage ccRCC tumors carry a high risk of venous invasion and that the presence of VTTs is strongly correlated with a poor prognosis in ccRCC patients. We therefore compared KRT8 expression levels in VTTs, tumors, and peritumor tissues. As shown in Figure 1E and 1F, KRT8 mRNA and protein levels were significantly higher in VTT tissues than in corresponding tumor or peritumoral tissues, suggesting that KRT8 may play an oncogenic role in ccRCC progression.

### KRT8 upregulation is associated with aggressive clinicopathological traits

To further investigate the clinical significance of KRT8 expression in ccRCC development and progression, we divided all 189 ccRCC patients into the following 2 groups based on their median KRT8 expression levels: a high KRT8 expression group (n=95) and a low KRT8 expression group (n=94). As shown in Table 1, KRT8 up-regulation in ccRCC tissues was significantly correlated with positive metastasis (p=0.039), higher pT stages (p=0.014), advanced clinical stages (p<0.001) and worse Fuhrman grades (p=0.026). We then examined KRT8 expression in ccRCC tissues with or without metastasis and determined the relationships between these expression levels and different ccRCC clinicopathological parameters. We noted upregulated KRT8 expression in ccRCC tissues with metastasis (Supplmentary Figure 1A) and with advanced clinic stages (III-IV) (Supplmentary Figure 1B), higher pT stages (III-IV) (Supplmentary Figure 1C) and worse Fuhrman grades (III-IV) (Supplmentary Figure 1D). These results showed that KRT8 upregulation was associated with multiple characteristics related to ccRCC aggression.

**Table 1:** Clinical characteristics of the 189 ccRCC patients according to their KRT8 expression levels

Variables	Patients (n)	the KRT8 expression		χ^2^	P value
		High expression	Low expression		
Gender					
Female	63	29	34	0.677	0.411
Male	126	66	60		
Age (years)					
<60	125	64	61	0.129	0.719
≥60	64	31	33		
BMI					
<25	128	65	63	0.042	0.837
≥25	61	30	31		
Laterality					
Right	97	47	50	0.261	0.609
Left	92	48	44		
pT stage					
pT1+pT2	140	63	77	5.987	0.014*
pT3+pT4	49	32	17		
Clinical stage					
I-II	115	46	69	12.379	<0.001*
III-IV	74	49	25		
Fuhrman grade					
I-II	122	54	68	4.960	0.026*
III-IV	67	41	26		
Tumor size (cm)					
≤7	104	47	57	2.380	0.123
>7	85	48	37		
Metastatic status					
Absent	147	68	79	4.247	0.039*
Present	42	27	15		

### KRT8 upregulation serves as a prognostic factor for patients with ccRCC

**Figure 2:**
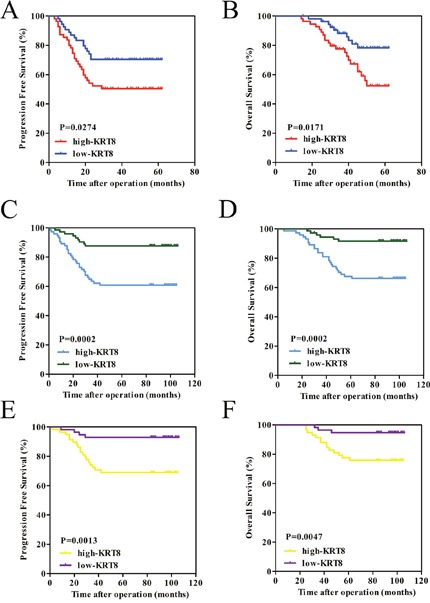
KRT8 upregulation serves as a prognostic factor for patients with ccRCC **(A** and **B)** The high KRT8 subgroup had significantly shorter PFS and OS than the low KRT8 subgroup in the qRT-PCR cohort. **(C** and **D)** Similar results were observed in the TMA cohort comprising 147 primary non-metastatic patients with ccRCC. **(E** and **F)** The prognostic value of KRT8 was also observed in patients with early stage localized ccRCC (pT stage I and II). Statistical significance was assessed by two-sided log-rank tests.

To determine the prognostic value of KRT8 in ccRCC, we generated Kaplan-Meier survival curves and performed log-rank tests in both the qRT-PCR and the TMA cohorts. The median expression level in each group was used as the cutoff value. Remarkably, we found that patients with higher KRT8 mRNA levels had significantly shorter PFS (p=0.0274) and OS (p=0.0171) than patients with lower KRT8 mRNA levels (Figure 2A and 2B). Moreover, we noted similar results in the TMA for the cohort comprising 147 primary non-metastatic tumor samples (p=0.0002 for PFS, p=0.0002 for OS) (Figure 2C and 2D). The Cox proportional hazards regression analysis results indicated that KRT8 expression levels were an independent risk factor for PFS (hazard ratio [HR] 2.918, 95 % confidence interval [CI] 1.342–6.345, p=0.007) and OS (HR 3.512, 95 % CI 1.391-8.867, p=0.008) in patients with ccRCC after partial or radical nephrectomy (Table 2). In our clinical experience, though the localized ccRCC cases of stage T1–T2 are considered clinically low risk, there still exists the possibility of metastasis. We analyzed the 115 localized ccRCC tissue samples of stage T1-T2 in the TMA cohort to investigate the value of KRT8 with respect to the determination of the early prognoses of these tumors further. Notably, we found that the prognosis-predictive value of KRT8 in early stage localized ccRCC (pT stages I and II) was significant (p=0.0013 for PFS, p=0.0047 for OS) (Figure 2E and 2F). Collectively, our findings indicated that KRT8 can be used as a predictor of prognosis in ccRCC patients after partial or radical nephrectomy, especially in patients with early stage localized ccRCC (pT stages I and II).

**Table 2:** Univariate and multivariate analyses of progression free survival and overall survival in patients with ccRCC

	Progression free survival (PFS)						Overall survival (OS)					
	Univariate analysis			Multivariate analysis			Univariate analysis			Multivariate analysis		
	Risk ratio	95% CI	P value	Risk ratio	95% CI	P value	Risk ratio	95% CI	P value	Risk ratio	95% CI	P value
Gender												
Male VS. Female	1.403	0.681-2.888	0.358				1.452	0.650-3.247	0.363			
Age (years)												
≥60 vs. <60	1.056	0.540-2.064	0.873				1.164	0.557-2.429	0.687			
BMI												
≥25 vs. <25	0.893	0.450-1.769	0.745				0.937	0.441-1.991	0.866			
Laterality												
Left vs. Right	0.990	0.524-1.872	0.976				0.717	0.348-1.476	0.366			
pT stage												
pT3+pT4 vs. pT1+pT2	3.578	1.875-6.825	<0.001*	2.612	1.337-5.103	0.005*	4.011	1.974-8.152	<0.001*	2.753	1.319-5.747	0.007*
Fuhrman grade												
III-IV vs. I-II	1.789	0.933-3.430	0.080				1.313	0.618-2.789	0.477			
Tumor size (cm)												
>7 vs. ≤7	1.704	0.895-3.247	0.105				1.291	0.638-2.612	0.477			
KRT8 levels												
High vs. Low	3.722	1.761-7.868	0.001*	2.918	1.342-6.345	0.007*	4.652	1.908-11.344	0.001*	3.512	1.391-8.867	0.008*

### KRT8 promotes ccRCC metastasis *in vitro*

**Figure 3:**
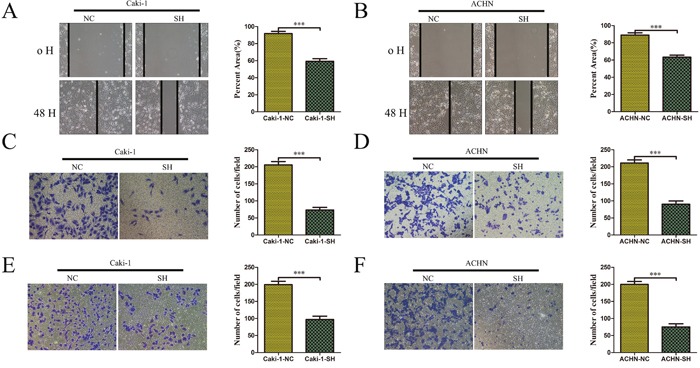
KRT8 promotes ccRCC metastasis *in vitro* Scratch wound healing assays and transwell assays showed that KRT8 knockdown inhibited the migratory and invasive properties of the renal cancer cell lines Caki-1 **(A, C** and **E)** and ACHN **(B, D** and **F)**. The representative results and statistical analysis are shown.

To explore the biological functions of KRT8 in ccRCC *in vitro*, we first detected KRT8 expression levels in several renal cancer cell lines (Supplmentary Figure 2A). We selected Caki-1, ACHN, and 786-O cells to generate stable KRT8-knockdown or overexpression cells (which we named Caki-1-SH, ACHN-SH, and 786-O-KRT8) using recombinant lentiviruses containing shRNAs targeting KRT8 or full-length KRT8. KRT8 knockdown or overexpression efficiency was confirmed by qRT-PCR and western blotting (Supplmentary Figure 2B-2D). In the wound healing migration assay, microscopic examination at 0 and 48 h showed that Caki-1-SH migration was significantly delayed compared with Caki-1-NC migration (Figure 3A). Transwell assay also revealed that Caki-1-SH cells displayed decreased migration and invasiveness compared with other cells (Figure 3C and 3E). Similar results were observed for the ACHN cell lines (Figure 3B, 3D and 3F). We also found that KRT8 overexpression could enhance 786-O-KRT8 cell migration and invasiveness (Supplmentary Figure 3A-3C). Additionally, CCK-8 assay revealed that KRT8 did not influence renal cancer cell growth (Supplmentary Figure 3D-3F). These data indicated that KRT8 may promote renal cancer cell metastasis *in vitro*.

### KRT8 promotes ccRCC metastasis *in vivo*

**Figure 4:**
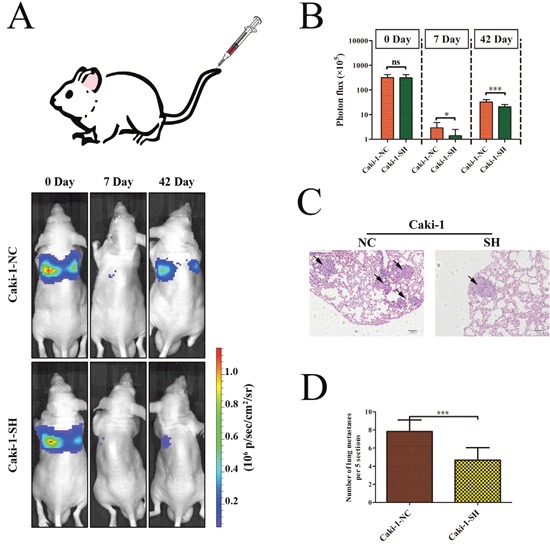
KRT8 promotes ccRCC metastasis *in vivo* **(A)** Images of lung metastases that developed in the Caki-1 cell lines in the lateral tail vein injection models. The images were acquired using an IVIS Imaging System. Representative luciferase signals captured in each group at the time of the initial injection: 0 days, 7 days and 42 days after cell injection are shown. The statistical analysis is shown in **(B)**. **(C)** Representative H&E-stained images of lung metastatic loci from each group in (A). The statistical analysis is shown in **(D)**.

To verify the function of KRT8 *in vivo*, we injected Caki-1-NC and Caki-SH cells directly into the tail veins of nude mice to establish a lung metastasis animal model. Since Caki-1-NC and Caki-1-SH express firefly luciferase, we dynamically monitored the process of lung metastasis for 0, 7, and 42 days using an *in vivo* imaging system. The results of the photon flux indicated that KRT8 knockdown inhibited lung metastasis (Figure 4A and 4B). After 42 days, the lungs were dissected and stained with H&E. We found that the lungs in the control group showed significantly more micrometastases compared with the lungs in the other group (Figure 4C and 4D). These data indicated that KRT8 can promote ccRCC metastasis *in vivo*.

### KRT8 activates IL-11/STAT3 signaling in ccRCC

**Figure 5:**
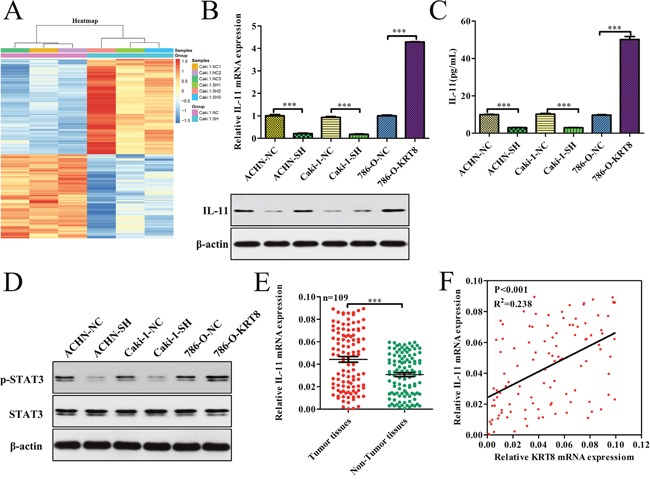
KRT8 activates IL-11/STAT3 signaling in ccRCC **(A)** Genes that are differentially expressed in Caki-1-SH cells compared with Caki-1-NC cells. **(B)** Relative IL-11 mRNA and protein expression levels in KRT8-knockdown cells, KRT8-overexpression cells and paired controls. IL-11 expression was analyzed by western blotting and normalized to β-actin. **(C)** IL-11 concentrations in the culture media from stable KRT8-knockdown or KRT-overexpression cells and paired controls were determined by ELISA. **(D)** Relative p-STAT3 and STAT3 expression levels in stable KRT8-knockdown cells, KRT8-overexpression cells and paired controls were analyzed using western blotting and were normalized to β-actin. **(E)** IL-11 mRNA expression levels in paired ccRCC tissue samples (n=109). **(F)** The correlation between KRT8 mRNA levels and IL-11 mRNA levels was measured in the same set of ccRCC tissues that was assessed in (E).

To explore the molecular mechanisms underlying KRT8-mediated increases in ccRCC metastasis, we first assessed whether KRT8 was involved in manipulating epithelial mesenchymal transition (EMT), which is thought to be a key process underlying cell metastasis. We found that KRT8 had no significant effect on the expression levels of EMT-related genes, such as E-cadherin, N-cadherin, and vimentin (Supplmentary Figure 4A-4D). To identify the genes potentially involved in enhancing KRT8 expression in renal cancer cell metastasis, we analyzed the resultant Caki-1-SH cell expression profiles using a gene microarray platform and subsequently included differentially expressed genes in functional classification analyses. A total of 161 genes were upregulated significantly, and 142 genes were down-regulated (>1.5-fold, SH/NC) in Caki-1-SH cells (Figure 5A). We found that the IL-11 was one of the top ten down-regulated genes in the down-regulated gene subgroup. Given that previous studies [26, 27] have reported that IL-11/STAT3 can regulate metastasis, we reasoned that KRT8 may promote metastatic cell survival through IL-11/STAT3 signaling activation. To further validate the effects of KRT8 on IL-11/STAT3 signaling *in vitro*, we measured IL-11 mRNA and protein levels in different ACHN, Caki-1, and 786-O cell clones and found that KRT8 knockdown significantly decreased IL-11 mRNA and protein levels in ACHN-SH and Caki-1-SH cells. In contrast, KRT8 overexpression increased IL-11 mRNA and protein levels in 786-O-KRT8 cells (Figure 5B). To examine whether KRT8 increases IL-11 secretion and activates IL-11/STAT3 signaling, we measured IL-11 levels in cell supernatants and STAT3 phosphorylation levels in different clones. KRT8 knockdown significantly decreased IL-11 levels in the cell supernatants and STAT3 phosphorylation levels in ACHN-SH and Caki-1-SH cells, while KRT8 overexpression increased IL-11 levels in the cell supernatants and STAT3 phosphorylation levels in 786-O-KRT8 cells (Figure 5C and 5D). To confirm that IL-11 is regulated by KRT8 in human ccRCC tissues, we measured IL-11 mRNA levels in the above mentioned set of 109 pairs of tumor and matched primary NT tissues, as shown in Figure 1A, and found that IL-11 mRNA levels were significantly higher in the tumor tissues than in the primary NT tissues (Figure 5E). Importantly, IL-11 mRNA levels were correlated with KRT8 transcript levels in tumor tissues (Figure 5F). All of these data indicate that KRT8 may increase IL-11 expression and activate IL-11/STAT3 signaling in ccRCC.

### KRT8 is required for the IL-11-mediated metastatic phenotype in ccRCC

**Figure 6:**
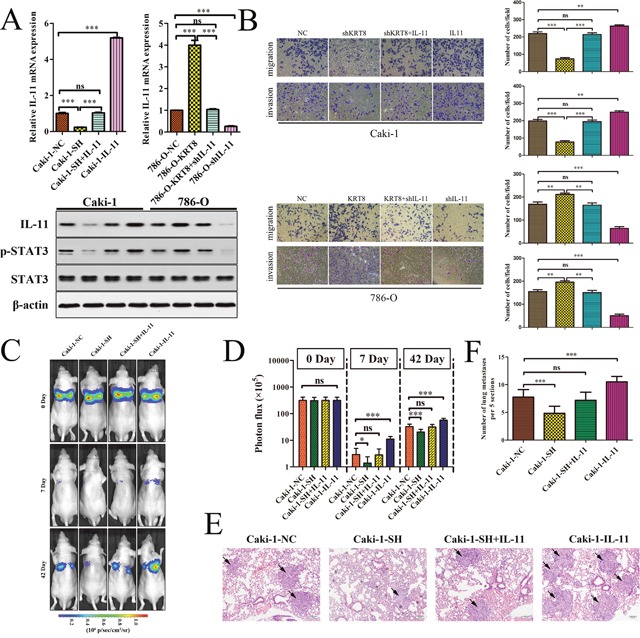
KRT8 is required for the IL-11-mediated metastatic phenotype in ccRCC **(A)** IL-11 mRNA levels were determined by qRT-PCR, and IL-11, p-STAT3, and STAT3 protein levels were determined by western blotting. **(B)** IL-11 overexpression restored Caki-1-SH-mediated migration and invasion ability. In contrast, IL-11 knockdown significantly reduced 786-O-KRT8 cell migration and invasion. **(C)** Representative images of the mice over time after the tail vein injections with each type of renal cancer cell. The statistical analysis is shown in **(D)**. **(E)** Representative H&E-stained images of the lung metastatic loci in each group in (C). The statistical analysis is shown in **(F)**.

To further explore whether KRT8 promotes renal cancer cell migration and invasion through IL-11/STAT3, we overexpressed or knocked down IL-11 to conduct functional studies in renal cancer cells (Figure 6A). IL-11 overexpression attenuated the effects of KRT8 knockdown on renal cancer cell migration and invasion (Figure 6B). Consistent with this finding, IL-11 knockdown abrogated the effects of KRT8 overexpression on renal cancer cell migration and invasion (Figure 6B). We further explored the roles of KRT8 and IL-11 in lung colonization by injecting cells directly into the tail veins of nude mice. We found that KRT8 knockdown cells showed decreased lung colonization and formed less micrometastatic lesion in the lung at the late time point; however, IL-11 overexpression largely attenuated the decrease in colonization rates (Figure 6C–6F). These data indicate that KRT8 is required for the IL-11-mediated metastatic phenotype in ccRCC.

## DISCUSSION

Partial nephrectomy is recommended as the standard treatment for localized ccRCC [28]; however, cancer metastasis is a severe problem in clinical treatment, warrants a significant change in therapeutic strategies and signifies poor outcomes in ccRCC [3, 29]. Once a tumor has metastasized, the mortality burden faced by ccRCC patients is significant [4]. Thus, there is an urgent need to determine which patients at high risk for developing metastasis may benefit from radical nephrectomy and adjuvant treatment. In addition, currently, cytokine therapy with IFN-a and IL-2 and targeted therapy including sunitinib and temsirolimus are the main treatments for metastatic ccRCC patients. Unfortunately, because of acquired resistance and other drawbacks, both therapies for metastatic ccRCC have limited efficacy and remain unsatisfactory with respect to patient outcomes [8, 30, 31]. Therefore, gaining a better understanding of the molecular mechanisms underlying ccRCC metastasis may enable researchers to identify reliable biomarkers to clinically diagnose affected patients, predict the prognoses of these patients and target therapies to treat these patients.

Though existing studies have identified that KRT8 expression is clinically significant in various cancers, the function and precise mechanisms of KRT8 in ccRCC are require further research. In this study, we found that KRT8 expression was significantly upregulated in ccRCC tissues comparison to adjacent renal normal tissues, and even further increased in VTTs. It was also shown that KRT8 upregulation was closely associated with several malignant clinical parameters in patients with ccRCC. Since it was previously reported that IL-6 could upregulate mRNA and protein levels of KRT8, it may explain the mechanism by which KRT expression was significantly increased in ccRCC [32].

Prognostic analyses showed that patients with higher KRT8 expression levels had poorer PFS and OS than patients with lower KRT8 expression levels. Furthermore, multivariate analysis revealed that KRT8 expression levels were an independent risk factor for both PFS and OS. Interestingly, our analysis of the association between intratumoral KRT8 protein expression and clinicopathologic features revealed the existence of a significant positive correlation between protein expression and metastasis status, pT stage, clinical stage and Fuhrman grade, all of which are hallmarks for a poor prognosis in ccRCC. These results suggest that KRT8 can be used as a biomarker for aggressive ccRCC and that it may be involved in ccRCC progression.

Generally, clinicians recommend that localized stage T1–T2 ccRCC cases, which are considered clinically low risk, are treated via partial or radical nephrectomy. Metastasis results in a poor outcome in some clinically low-risk ccRCC cases. Thus, identifying ccRCC prognostic and metastasis risk markers, especially at stage T1–T2, is important. Here, further prognostic analyses revealed that early stage localized ccRCC (T1–T2) with higher KRT8 expression also had poorer PFS and OS than early stage localized disease with lower KRT8 expression. Therefore, our current results suggest that KRT8 may serve as a promising prognostic biomarker with which ccRCC patients can be stratified into distinct risk subgroups to receive appropriate disease-monitoring and guided individualized therapy.

The significant correlations between KRT8 expression levels in tumors and aggressive clinical behavior and poor ccRCC patient prognoses prompted us to investigate whether KRT8 plays a functional role in ccRCC invasion and metastasis. We subsequently found that silencing KRT8 using specific shRNAs decreased renal cancer cell invasion and metastasis abilities, whereas KRT8 overexpression led to significant increases in ccRCC invasion and metastasis ability *in vitro*. Similar results were also observed *in vivo*. We also demonstrated that KRT8 did not affect renal cancer cell proliferation. These results also suggest that KRT8 may be a potential therapeutic target in ccRCC metastasis.

We explored the molecular mechanisms that potentially underlie ccRCC progression and metastasis further and found that the expression levels of EMT-related genes, such as E-cadherin, N-cadherin, and vimentin, were not significantly affected by KRT8, indicating that KRT8-mediated renal cancer cell metastasis is an EMT-independent process. To identify the genes that are potentially involved in enhancing KRT8 expression in renal cancer cell metastasis, we analyzed the resultant Caki-1-SH cell expression profile using a gene microarray platform. We identified 303 differentially expressed genes and then analyzed the expression profiles resulting from KRT8 knockdown. We observed that KRT8 down-regulation decreased IL-11 expression levels. A member of the IL-6 cytokine family, IL-11 has been found to play an oncogenic role in various malignancies, including gastric carcinoma [33], breast cancer [34], and hepatocellular carcinoma [35]. The results of previous studies also showed that RCC cells can produce IL-11 *in vitro* [36] and that high IL-11 expression is an independent predictor of a poor prognosis in ccRCC patients [37]. IL-11 transmits signals through its unique receptor, IL-11Rα, which couples with the common receptor subunit GP130 to activate the downstream STAT3 signaling pathway, a pathway that is closely associated with cancer pathogenesis [38]. Ren et al. [39] found that the elevated IL-11 expression can promote bone metastasis in breast cancer via the GP130/STAT3 pathway. And it was found that miR-30c significantly suppressed lung metastases of breast cancer cells by targeting TWF1 and regulating IL-11-pSTAT3 signaling pathway *in vivo* [40]. IL-11 facilitated lung cancer cell chemoresistance to cisplatin via the IL-11R/STAT3 signaling pathway by promoting activation of the anti-apoptotic proteins Bcl-2 and Survivin in lung adenocarcinoma [41]. As a crucial cytokine, IL-11 has been suggested to contribute to the promotion of chronic gastric inflammation and associated tumorigenesis via excessive STAT3 and STAT1 activation [42]. TMED3-mediated IL-11/STAT3 signaling pathway activation was recently reported to participate in tumor progression in hepatocellular carcinoma [43]. In addition, Yuan et al. reported that lncRNA-ATB could promote lung colonization of hepatocellular carcinoma tumor cells by triggering IL-11/STAT3 signaling pathway [44]. Considering the important role of IL-11/STAT3 signaling pathway in tumor progression and metastasis, therapeutic options using direct STAT3 inhibitors or upstream inhibitors might be used for the treatment of ccRCC in the future. As a novel inhibitor of GP130/STAT3 signaling, Bazedoxifene could suppress pancreatic cancer growth *in vitro* and *in vivo* and sensitize pancreatic cancer cells to paclitaxel and gemcitabine [45]. In addition, Winship et al. [46, 47] reported that therapeutically targeting IL11 Receptor-α could effectively inhibit endometrial cancer growth and dissemination by reducing STAT3 phosphorylation.

We combined western blotting and RT-PCR analysis to determine that KRT8 can increase IL-11 expression, cause IL-11 autocrine induction, and activate IL-11/STAT3 signaling, findings that were verified further in our *in vitro* system, our xenograft metastasis model, and clinical ccRCC tissues. The results of this experiment indicated that KRT8 may promote ccRCC metastasis through IL-11/STAT3 signaling. However, the relationship between KRT8 and IL-11 remains unclear and requires further investigation.

In conclusion, our results provide new evidence to support the idea that KRT8 expression levels were significantly higher in metastatic ccRCC tumor tissues than in non-metastatic ccRCC tumor tissues. High-level KRT8 expression may promote metastasis by activating IL-11/STAT3 signaling, however, the mechanism underlying KRT8 regulation in ccRCC awaits further investigation.

## MATERIALS AND METHODS

### Ethics statement

This study was approved by the Ethics Committee of the Second Military Medical University, and informed consent was obtained from all patients before the study was initiated. All animal experimental protocols were approved by the Institutional Animal Care and Use Committee of the Second Military Medical University, Shanghai, China.

### Patients and specimens

All cases included in the study were clinically and pathologically identified as ccRCC. This study included 109 ccRCC tissues and paired non-tumor (NT) tissues obtained from Changhai Hospital for quantitative real-time PCR (qRT-PCR) analysis. These tissues were used to determine KRT8 mRNA expression levels, and their clinicopathologic characteristic are shown in Supplementary Table 1. For further confirmation that KRT8 may be associated with tumor metastasis, we used additional samples from 15 patients with ccRCC and venous tumor thrombi to evaluate KRT8 expression levels in paired ccRCC/NT/VTT samples by qRT-PCR and immunohistochemical analysis. To study the correlations between KRT8 expression and clinical characteristics and prognosis, we studied a large cohort of 189 patients with ccRCC (randomly collected from February 2007 to September 2014) who received high-quality follow-up to examine KRT8 protein levels by tissue microarray (TMA) and IHC analysis and ascertain the clinical significance of KRT8. The clinicopathologic characteristics of these patients are shown in Table 1. All ccRCC specimens were obtained immediately after radical or partial nephrectomy, and the VTTs were obtained from the renal vein or vena cava with tumor thrombi extraction. And all the fresh specimens used in this study were immediately snap-frozen within liquid nitrogen, and stored at -80°C until further use. Clinical stages and pT stages were determined according to the 2011 Union for International Cancer Control (UICC) TNM classification guidelines.

### TMA construction and immunohistochemical analysis

Briefly, all the tissue samples obtained from patients in the ccRCC cohort were reviewed histologically using H&E staining. Representative areas distant from necrotic and hemorrhagic tissue were marked on the paraffin blocks. Two 1.0-mm cores were extracted from each tumor sample, paired with NT tissues and added to a new recipient block using a semi-automated arraying device (TMArrayer Pathology Devices, USA). IHC was performed on the TMA construction using a two-step immunoperoxidase technique. After heating the sections in 10 mmol/l citrate buffer for antigen retrieval, we incubated the sections with the primary antibody against KRT8 (Abcam, CA, USA; dilution 1:60) at 4 °C overnight and then incubated them with the appropriate secondary antibody for one hour at room temperature. Subsequently, the sections were developed by adding diaminobenzidine solution and counterstained with hematoxylin. The IHC stains were assessed by three separate pathologists who were blinded to patient characteristics.

The photographs of all slides were captured under high power view with Leica QWin Plus v3 software. Density of KRT8 positive staining was quantifed using Leica DFC420 CCD camera connected to Leica DM IRE2 microscope (Leica Microsystems Imaging Solutions Ltd, Cambridge, United Kingdom). With the same setting for the background of the photographs, the integrated optical density of KRT8 was counted with the ImagePro Plus v6.0 software (Media Cybernetics Inc, MD, USA) as previously described [48]. KRT8 protein density of each photograph was calculated as the ratio of integrated optical density to total tissue area.

### Cell culture

The renal cancer cell lines 786-O, ACHN, A498, Caki-1 and human normal proximal tubule epithelial cell line HK-2 used in the study were purchased from the Shanghai Institute of Life Sciences Cell Resource Center (Shanghai, China). The cell lines were cultured in RPMI-1640 medium (HyClone), Dulbecco's modified Eagle medium (DMEM, HyClone), DMEM/F12 (HyClone), or McCoy's 5A Medium (HyClone) supplemented with 10 % fetal bovine serum (FBS) and 1 % penicillin/streptomycin (Gibco, USA), according to the American Type Culture Collection. All cell cultures were maintained at 37 °C in a humidified atmosphere with 5 % CO_2_.

### RNA extraction, cDNA preparation and qRT-PCR

Total RNA was extracted from cells in cultures and tissues cut from frozen specimens using TRIzol reagent (Takara, Japan), according to the manufacturer's instructions. Total RNA quality was assessed using a Nanodrop 2000 and agarose gel electrophoresis. First-strand cDNA was generated from 2 μg of total RNA using M-MLV reverse transcriptase (Invitrogen, CA) with random primers. qRT-PCR was performed according to the SYBR Green protocol in a Step One Plus System (Applied Biosystems, Foster City, CA, USA), and β-actin served as the endogenous control. The specific sequences of primers used for the PCR reaction are shown in Supplementary Table 2. Relative mRNA expression levels were calculated based on the corresponding Ct values and were normalized to β-actin expression.

### Western blot analysis

Total cell and tissue lysates were prepared in 1× sodium dodecyl sulfate buffer. Identical quantities of protein were separated by SDS-gel electrophoresis and transferred onto nitrocellulose filter membranes. After incubating with antibodies specific for KRT8 (ab216022, ab215830; Abcam, CA, USA), IL-11 (ab187167; Abcam, CA, USA), STAT3 (ab119352; Abcam, CA, USA), P-STAT3 (ab76315; Abcam, CA, USA) and β-actin (ab8226; Abcam, CA, USA), the blots were incubated with IRDye 800-conjugated goat anti-rabbit IgG and IRDye 700-conjugated goat anti-mouse IgG, and the bands were detected using an Odyssey infrared scanner (Li-Cor). β-Actin was used as the loading control.

### Wound healing migration assay

Briefly, 1 × 10^5^ cells/well were plated in 6-well plates. After the cells attached to the plates, a wound was created in the middle of each well, and the medium was replaced with serum-free medium. The area of healing across the lesion was measured after a 48-h incubation period.

### Transwell assays

The 24-well culture insert plates (Millipore, USA) and polycarbonate membranes with a pore size of 8 μm were used for transwell assays. Firstly, the insert plates were equilibrated with 0.5 ml of serum-free culture medium for 1 h at 37 °C in 5 % CO_2_. Then, the medium in the lower chambers was replaced with 0.5 ml of culture medium supplemented with 10 % FBS. 5×10^4^ cells serum pre-starved renal cancer cells in 400 μl serum free media were seeded into the upper chambers. After a 48-h incubation period, the inserts were rinsed with PBS, and the cells on the upper surface of the membrane were scraped off. The cells on the bottom side of the membrane were stained with crystal violet stain and counted by a microscope. Cells from each culture condition were examined in quadruplicate.

### Cell counting kit 8 (CCK8) assay

Renal cancer cells were cultured for 12, 24, 36 and 48 h. The wells to which only culture medium was added served as blanks. At different time point, the supernatant was removed, and 100 μl of culture medium containing 10 μl of CCK8 was added to each well for another 2 h of incubation at 37 °C. The absorbance was recorded at 450 nm. All experiments were independently repeated in triplicate on separate occasions.

### Animal studies

To explore the effects of KRT8 on ccRCC metastasis *in vivo*, the lateral tail vein injection model was used to evaluate the potential of the tumor cells to metastasize to the lungs. The metastases of the lungs were monitored using an IVIS@ Lumina II system (CaliperLife Sciences, Hopkinton, MA, USA) for 10 min after intraperitoneal injection of 4.0 mg of luciferin (Gold Biotech) in 50 μl of saline. The nude mice were housed in cages under standard conditions, and the experiments were performed according to the requirements of the Second Military Medical University Animal Care Facility and the National Institutes of Health guidelines. The mice were maintained in pathogen-free conditions.

### Gene expression profiling and analysis

Total RNA was extracted using TRIZOL reagent (Cat#15596-018, Life Technologies, Carlsbad, CA, USA) according to the manufacturer's instructions, and was checked for RNA Integrity Number (RIN) to using an Agilent 2100 Bioanalyzer (Agilent Technologies, Santa Clara, CA, USA). Qualified total RNA was further purified using an RNeasy Micro Kit (Cat#74004, QIAGEN, GmBH, Germany) and RNase-Free DNase Set (Cat#79254, QIAGEN, GmBH, Germany). Comparative microarray analysis of Caki-1-NC or Caki-1-SH cell mRNA was performed on GeneChip^®^PrimeView^™^ Human Gene Expression arrays (Affymetrix), in accordance with a protocol developed by Shanghai Biotechnology Corporation. Briefly, total RNA was amplified, labeled and purified using a GeneChip 3' IVT PLUS Reagent Kit (Cat#902416, Affymetrix, Santa Clara, CA, USA), according to the manufacturer's instructions, to obtain biotin labeled cRNA. Array hybridization and washings were performed using a GeneChip^®^ Hybridization, Wash and Stain Kit (Cat#900720, Affymetrix, Santa Clara, CA, USA) in a Hybridization Oven 645 (Cat#00-0331-220V, Affymetrix, Santa Clara, CA, USA) and Fluidics Station 450 (Cat#00-0079, Affymetrix, Santa Clara, CA, USA), according to the manufacturer's instructions. Slides were scanned by a GeneChip^®^ Scanner 3000 (Cat#00-00212, Affymetrix, Santa Clara, CA, USA) and Command Console Software 4.0 (Affymetrix, Santa Clara, CA, USA) using default settings. The raw data were normalized by an RMA algorithm, Affy package, using R.control. Significant genes were filtered for detection using a fold change > 1.5, and p<0.05 (Student's t-test).

### IL-11 ELISA

IL-11 levels were detected in normal culture medium collected from different cells after 48 h using a Human IL-11 ELISA Kit (ab189569; Abcam, CA, USA), according to the manufacturer's instructions.

### Statistical analysis

Statistical analyses were performed using GraphPad Prism 5.0 and SPSS 18.0. Numerical data are presented as the mean and standard error. Differences between proportions were evaluated using paired Student's t-tests. The correlations between KRT8 expression and clinicopathologic parameters were evaluated using chi-square tests, and survival was evaluated via Kaplan–Meier analysis. P values < 0.05 were considered statistically significant.

## SUPPLEMENTARY MATERIALS FIGURES AND TABLES



## References

[1] Siegel RL, Miller KD, Jemal A (2016). Cancer statistics, 2016. CA Cancer J Clin.

[2] Rini BI, Campbell SC, Escudier B (2009). Renal cell carcinoma. Lancet.

[3] Ljungberg B, Bensalah K, Canfield S, Dabestani S, Hofmann F, Hora M, Kuczyk MA, Lam T, Marconi L, Merseburger AS, Mulders P, Powles T, Staehler M (2015). EAU guidelines on renal cell carcinoma: 2014 update. Eur Urol.

[4] Novara G, Ficarra V, Antonelli A, Artibani W, Bertini R, Carini M, Cosciani CS, Imbimbo C, Longo N, Martignoni G, Martorana G, Minervini A, Mirone V (2010). Validation of the 2009 TNM version in a large multi-institutional cohort of patients treated for renal cell carcinoma: are further improvements needed. Eur Urol.

[5] Jun G, Manuel CM, Nazli D, Ameish G, Sumanta KP (2016). Metastasis in renal cell carcinoma: biology and implications for therapy. Asian J Urol.

[6] Pichler M, Hutterer GC, Chromecki TF, Jesche J, Kampel-Kettner K, Rehak P, Pummer K, Zigeuner R (2011). External validation of the Leibovich prognosis score for nonmetastatic clear cell renal cell carcinoma at a single European center applying routine pathology. J Urol.

[7] Huang QB, Ma X, Li HZ, Ai Q, Liu SW, Zhang Y, Gao Y, Fan Y, Ni D, Wang BJ, Zhang X (2014). Endothelial Delta-like 4 (DLL4) promotes renal cell carcinoma hematogenous metastasis. Oncotarget.

[8] Gupta K, Miller JD, Li JZ, Russell MW, Charbonneau C (2008). Epidemiologic and socioeconomic burden of metastatic renal cell carcinoma (mRCC): a literature review. Cancer Treat Rev.

[9] Linehan WM, Bratslavsky G, Pinto PA, Schmidt LS, Neckers L, Bottaro DP, Srinivasan R (2010). Molecular diagnosis and therapy of kidney cancer. Annu Rev Med.

[10] Fuchs E, Weber K (1994). Intermediate filaments: structure, dynamics, function, and disease. Annu Rev Biochem.

[11] Coulombe PA, Omary MB (2002). 'Hard' and 'soft' principles defining the structure, function and regulation of keratin intermediate filaments. Curr Opin Cell Biol.

[12] Hesse M, Zimek A, Weber K, Magin TM (2004). Comprehensive analysis of keratin gene clusters in humans and rodents. Eur J Cell Biol.

[13] Schweizer J, Bowden PE, Coulombe PA, Langbein L, Lane EB, Magin TM, Maltais L, Omary MB, Parry DA, Rogers MA, Wright MW (2006). New consensus nomenclature for mammalian keratins. J Cell Biol.

[14] Toivola DM, Boor P, Alam C, Strnad P (2015). Keratins in health and disease. Curr Opin Cell Biol.

[15] Karantza V (2011). Keratins in health and cancer: more than mere epithelial cell markers. Oncogene.

[16] van Sprundel RG, van den Ingh TS, Desmet VJ, Katoonizadeh A, Penning LC, Rothuizen J, Roskams T, Spee B (2010). Keratin 19 marks poor differentiation and a more aggressive behaviour in canine and human hepatocellular tumours. Comp Hepatol.

[17] Li Q, Yin L, Jones LW, Chu GC, Wu JB, Huang JM, Li Q, You S, Kim J, Lu YT, Mrdenovic S, Wang R, Freeman MR (2016). Keratin 13 expression reprograms bone and brain metastases of human prostate cancer cells. Oncotarget.

[18] Escobar-Hoyos LF, Yang J, Zhu J, Cavallo JA, Zhai H, Burke S, Koller A, Chen EI, Shroyer KR (2014). Keratin 17 in premalignant and malignant squamous lesions of the cervix: proteomic discovery and immunohistochemical validation as a diagnostic and prognostic biomarker. Mod Pathol.

[19] Hobbs RP, Batazzi AS, Han MC, Coulombe PA (2016). Loss of keratin 17 induces tissue-specific cytokine polarization and cellular differentiation in HPV16-driven cervical tumorigenesis in vivo. Oncogene.

[20] Obermajer N, Doljak B, Kos J (2009). Cytokeratin 8 ectoplasmic domain binds urokinase-type plasminogen activator to breast tumor cells and modulates their adhesion, growth and invasiveness. Mol Cancer.

[21] Lau AT, Chiu JF (2007). The possible role of cytokeratin 8 in cadmium-induced adaptation and carcinogenesis. Cancer Res.

[22] Gires O, Mack B, Rauch J, Matthias C (2006). CK8 correlates with malignancy in leukoplakia and carcinomas of the head and neck. Biochem Biophys Res Commun.

[23] Matthias C, Mack B, Berghaus A, Gires O (2008). Keratin 8 expression in head and neck epithelia. BMC Cancer.

[24] Fang J, Wang H, Liu Y, Ding F, Ni Y, Shao S (2017). High KRT8 expression promotes tumor progression and metastasis of gastric cancer. Cancer Sci.

[25] Trisdale SK, Schwab NM, Hou X, Davis JS, Townson DH (2016). Molecular manipulation of keratin 8/18 intermediate filaments: modulators of FAS-mediated death signaling in human ovarian granulosa tumor cells. J Ovarian Res.

[26] Wang DQ, Ding XP, Yin S, Mao YD (2016). Role of the IL-11/STAT3 signaling pathway in human chronic atrophic gastritis and gastric cancer. Genet Mol Res.

[27] Buzzelli JN, Pavlic DI, Chalinor HV, O'Connor L, Menheniott TR, Giraud AS, Judd LM (2015). IL-1RT1 signaling antagonizes IL-11 induced STAT3 dependent cardiac and antral stomach tumor development through myeloid cell enrichment. Oncotarget.

[28] Campbell SC, Novick AC, Belldegrun A, Blute ML, Chow GK, Derweesh IH, Faraday MM, Kaouk JH, Leveillee RJ, Matin SF, Russo P, Uzzo RG (2009). Guideline for management of the clinical T1 renal mass. J Urol.

[29] Cohen HT, McGovern FJ (2005). Renal-cell carcinoma. N Engl J Med.

[30] Motzer RJ, Bacik J, Schwartz LH, Reuter V, Russo P, Marion S, Mazumdar M (2004). Prognostic factors for survival in previously treated patients with metastatic renal cell carcinoma. J Clin Oncol.

[31] Weinstock M, McDermott D (2015). Targeting PD-1/PD-L1 in the treatment of metastatic renal cell carcinoma. Ther Adv Urol.

[32] Wang L, Srinivasan S, Theiss AL, Merlin D, Sitaraman SV (2007). Interleukin-6 induces keratin expression in intestinal epithelial cells: potential role of keratin-8 in interleukin-6-induced barrier function alterations. J Biol Chem.

[33] Nakayama T, Yoshizaki A, Izumida S, Suehiro T, Miura S, Uemura T, Yakata Y, Shichijo K, Yamashita S, Sekin I (2007). Expression of interleukin-11 (IL-11) and IL-11 receptor alpha in human gastric carcinoma and IL-11 upregulates the invasive activity of human gastric carcinomacells. Int J Oncol.

[34] Johnstone CN, Chand A, Putoczki TL, Ernst M (2015). Emerging roles for IL-11 signaling in cancer development and progression: focus on breast cancer. Cytokine Growth Factor Rev.

[35] Gao YB, Xiang ZL, Zhou LY, Wu ZF, Fan J, Zeng HY, Zeng ZC (2013). Enhanced production of CTGF and IL-11 from highly metastatic hepatoma cells under hypoxic conditions: an implication of hepatocellular carcinoma metastasis to bone. J Cancer Res Clin Oncol.

[36] Knoefel B, Nuske K, Steiner T, Junker K, Kosmehl H, Rebstock K, Reinhold D, Junker U (1997). Renal cell carcinomas produce IL-6, IL-10, IL-11, and TGF-beta 1 in primary cultures and modulate T lymphocyte blast transformation. J Interferon Cytokine Res.

[37] Pan D, Xu L, Liu H, Zhang W, Liu W, Liu Y, Fu Q, Xu J (2015). High expression of interleukin-11 is an independent indicator of poor prognosis in clear-cell renal cell carcinoma. Cancer Sci.

[38] Grivennikov SI (2013). IL-11: a prominent pro-tumorigenic member of the IL-6 family. Cancer Cell.

[39] Ren L, Wang X, Dong Z, Liu J, Zhang S (2013). Bone metastasis from breast cancer involves elevated IL-11 expression and the gp130/STAT3 pathway. Med Oncol.

[40] Bockhorn J, Yee K, Chang YF, Prat A, Huo D, Nwachukwu C, Dalton R, Huang S, Swanson KE, Perou CM, Olopade OI, Clarke MF, Greene GL (2013). MicroRNA-30c targets cytoskeleton genes involved in breast cancer cell invasion. Breast Cancer Res Treat.

[41] Tao L, Huang G, Wang R, Pan Y, He Z, Chu X, Song H, Chen L (2016). Cancer-associated fibroblasts treated with cisplatin facilitates chemoresistance of lung adenocarcinoma through IL-11/IL-11R/STAT3 signaling pathway. Sci Rep.

[42] Ernst M, Najdovska M, Grail D, Lundgren-May T, Buchert M, Tye H, Matthews VB, Armes J, Bhathal PS, Hughes NR, Marcusson EG, Karras JG, Na S (2008). STAT3 and STAT1 mediate IL-11-dependent and inflammation-associated gastric tumorigenesis in gp130 receptor mutant mice. J Clin Invest.

[43] Zheng H, Yang Y, Han J, Jiang WH, Chen C, Wang MC, Gao R, Li S, Tian T, Wang J, Ma LJ, Ren H, Zhou WP (2016). TMED3 promotes hepatocellular carcinoma progression via IL-11/STAT3 signaling. Sci Rep.

[44] Yuan JH, Yang F, Wang F, Ma JZ, Guo YJ, Tao QF, Liu F, Pan W, Wang TT, Zhou CC, Wang SB, Wang YZ, Yang Y (2014). A long noncoding RNA activated by TGF-beta promotes the invasion-metastasis cascade in hepatocellular carcinoma. Cancer Cell.

[45] Wu X, Cao Y, Xiao H, Li C, Lin J (2016). Bazedoxifene as a novel GP130 inhibitor for pancreatic cancer therapy. Mol Cancer Ther.

[46] Winship AL, Van Sinderen M, Donoghue J, Rainczuk K, Dimitriadis E (2016). Targeting Interleukin-11 Receptor-alpha impairs human endometrial cancer cell proliferation and Invasion in vitro and reduces tumor growth and metastasis in vivo. Mol Cancer Ther.

[47] Winship A, Van Sinderen M, Rainczuk K, Dimitriadis E (2017). Therapeutically blocking Interleukin-11 Receptor-alpha enhances doxorubicin cytotoxicity in high grade type I endometrioid tumours. Oncotarget.

[48] Cai J, Yuan SX, Yang F, Tao QF, Yang Y, Xu QG, Wang ZG, Yu J, Lin KY, Wang ZY, Ma JZ, Zhou CC, Wang F (2016). Paraoxonase 3 inhibits cell proliferation and serves as a prognostic predictor in hepatocellular carcinoma. Oncotarget.

