# Supplementary material for: KRT8 upregulation promotes tumor metastasis and is predictive of a poor prognosis in clear cell renal cell carcinoma
Source: Oncotarget. 2017 Jul 12;8(44):76189–203. doi: 10.18632/oncotarget.19198 (PMC5652697; doi:10.18632/oncotarget.19198)
Supplement: Supplementary file 1 [file oncotarget-08-76189-s001.pdf]

KRT8 upregulation promotes tumor metastasis and is predictive of a poor prognosis in clear cell renal cell carcinoma

SUPPLEMENTARY MATERIALS

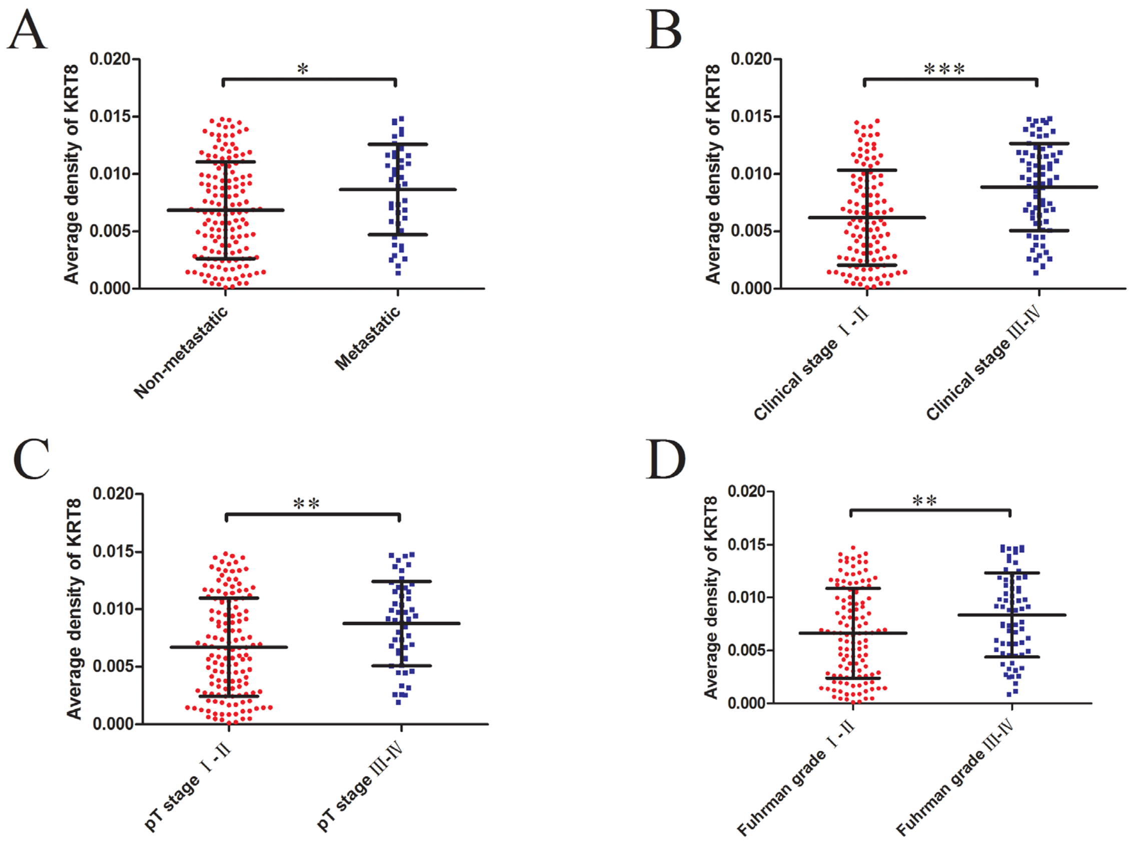

Supplementary Figure 1: (A-D) Correlations between KRT8 protein expression levels and ccRCC metastatic status, clinical stage, pT stage and Fuhrman grade, respectively.

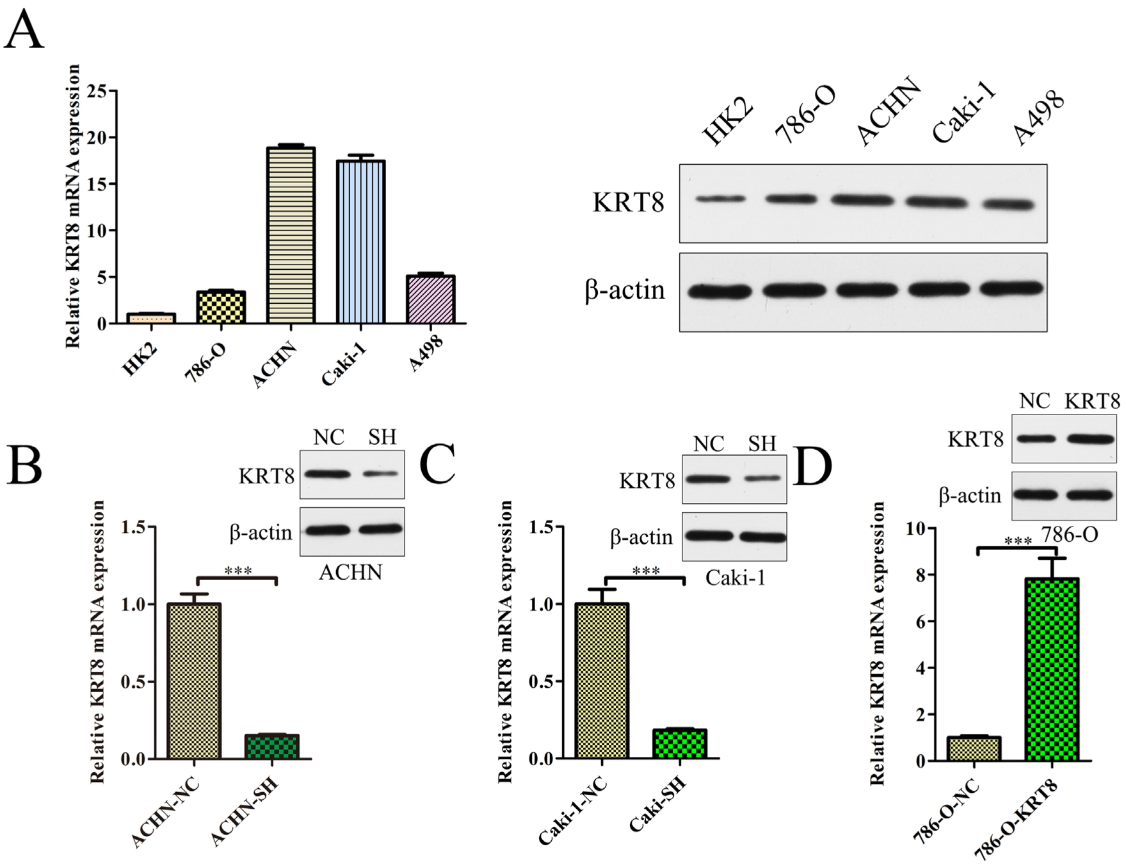

Supplementary Figure 2: (A) KRT8 mRNA and protein expression levels in multiple renal cancer cell lines and a normal renal cell line (HK-2). **(B-D)** The efficiency of KRT8 knockdown or overexpression in stably transfected cells. Data are shown as mean ± SD. Significance ($p<0.05$) was determined from three independent experiments and assessed using Student's t-test.

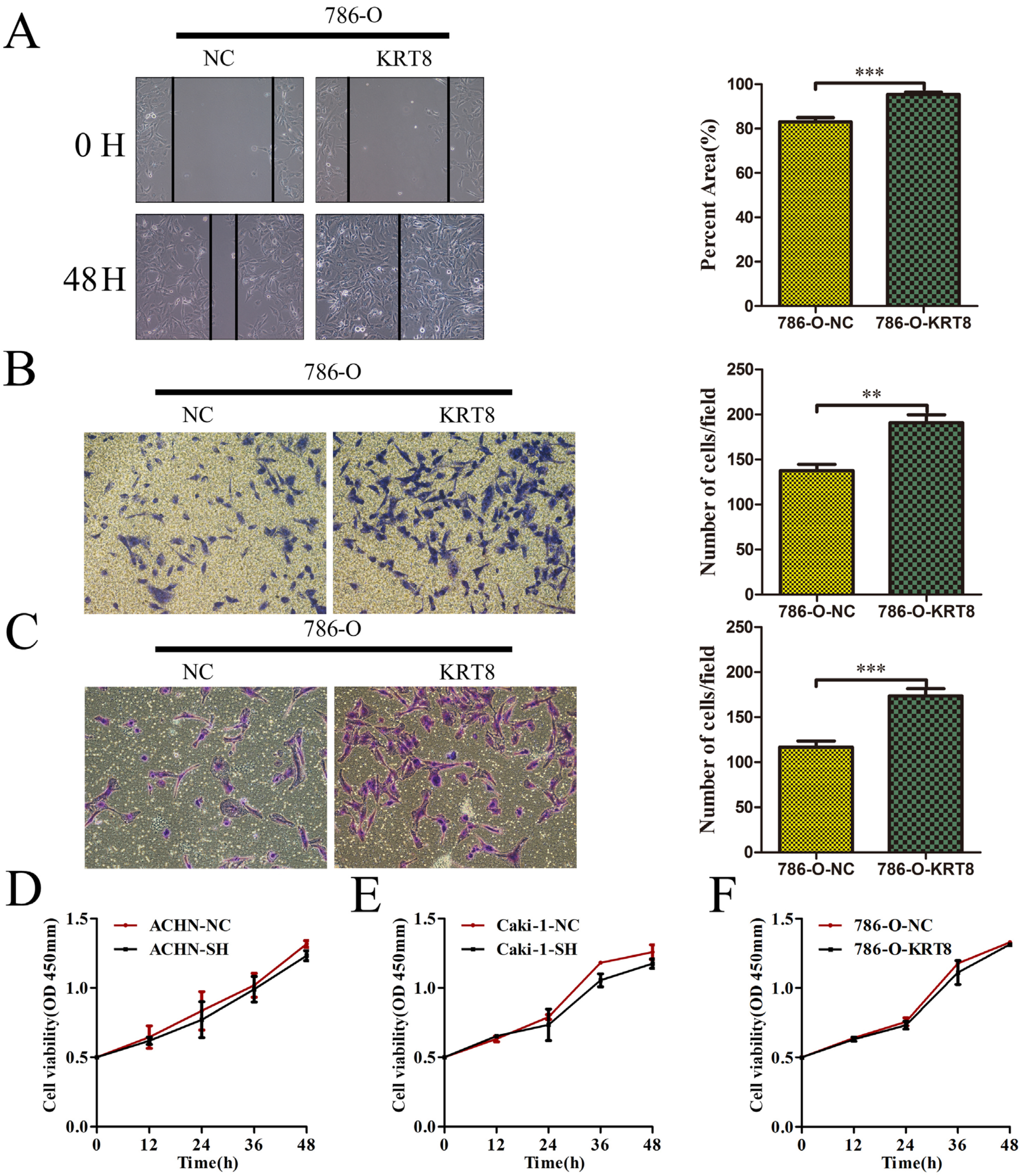

Supplementary Figure 3: (A) Scratch wound healing assays and **(B-C)** transwell assays showed that the KRT8 overexpression enhanced the migratory and invasive properties of the 786-O cell line. The representative results and statistical analysis are shown. **(D-F)** The growth curves of the ACHN-SH, Caki-SH, 786-O-KRT8 cell lines and their controls, respectively, in CCK8 assay.

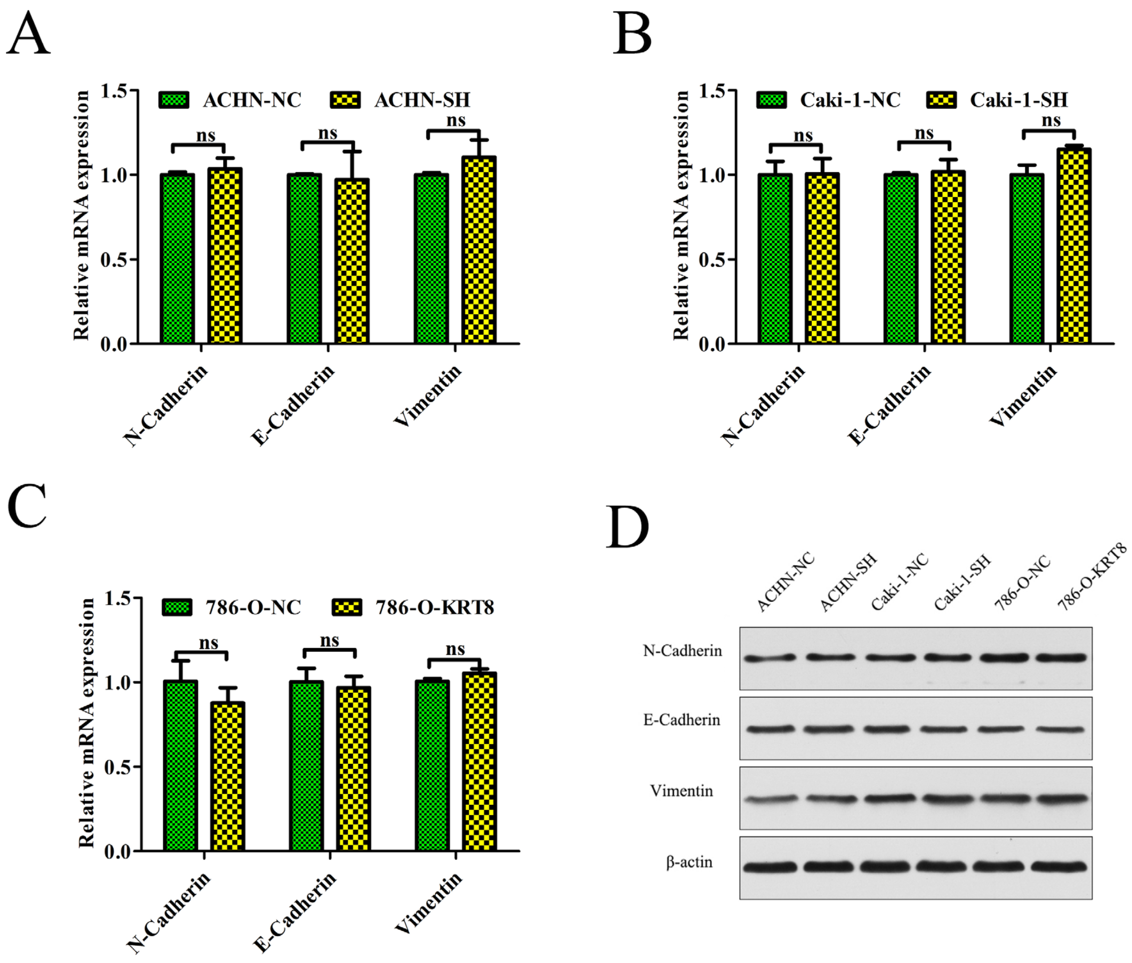

Supplementary Figure 4: (A-C) E-cadherin, N-cadherin, and vimentin mRNA levels were compared by RT-PCR, and **(D)** E-cadherin, N-cadherin, and vimentin protein levels were compared by western blot analysis.

Supplementary Table 1: Patient clinical characteristics

Clinicopathological parameters	Cohort 1 (n=109)	Cohort 2 (n=189)
	No. (%)	No. (%)
Gender		
Male	69 (63.3)	126 (66.7)
Female	40 (36.7)	63 (33.3)
Age (years)		
<60	59 (54.1)	125 (66.1)
≥60	50 (45.9)	64 (33.9)
Laterality		
Left	54 (49.5)	92 (48.7)
Right	55 (50.5)	97 (51.3)
BMI		
<25	66 (60.6)	128 (67.7)
≥25	43 (39.4)	61 (32.3)
Tumor size (cm)		
≤7cm	74 (67.9)	104 (55.0)
>7cm	35 (32.1)	85 (45.0)
pT stage		
T1-T2	82 (75.2)	140 (74.1)
T3-T4	27 (24.8)	49 (25.9)
Fuhrman grade		
I-II	71 (65.1)	122 (64.6)
III-IV	38 (34.9)	67 (35.4)

TNM staging: TNM stage groupings were assigned according to the 2009 TNM staging classification system
BMI, body mass index.

Supplementary Table 2: Sequences of the primers used for real-time PCR

KRT8, forward	TGGAGCAGCAGAACAAGATG
KRT8, reverse	CCGCCTAAGGTTGTTGATGT
IL-11, forward	GCTGCAAGGTCAAGATGGTT
IL-11, reverse	GCTGGGTGGCGTTCTATC
N-cadherin, forward	TGGACCATCACTCGGCTTA
N-cadherin, reverse	ACACTGGCAAACCTTCACG
E-cadherin, forward	GCCCCATCAGGCCTCCGTTT
E-cadherin, reverse	ACCTTGCCTTCTTTGTCTTTGTTGGA
Vimentin, forward	CCTGAACCTGAGGGAAACTAA
Vimentin, reverse	GCAGAAAGGCACTTGAAAGC
β-actin, forward	GGGAAATCGTGCGTGACATTAAG
β-actin, reverse	TGTGTTGGCGTACAGGTCTTTG
